# Influence of the environment on the characteristics of asthma

**DOI:** 10.1038/s41598-022-25028-1

**Published:** 2022-11-28

**Authors:** Christian Romero-Mesones, Iñigo Ojanguren, David Espejo, G. Granados, Francisco-Javier González-Barcala, María-Jesús Cruz, Xavier Muñoz

**Affiliations:** 1 Servicio de Neumología, Departamento de Medicina, Hospital Universitario Vall d’Hebron, Universidad Autónoma de Barcelona, Passeig Vall d’Hebron, 119 08035 Barcelona, Catalonia, Spain; 2 CIBER Enfermedades Respiratorias (Ciberes), Madrid, Spain; 3 Departamento de Biología Celular, Fisiología E Inmunología, Universidad Autónoma de Barcelona, Catalonia, Spain; 4 Servicio de Neumología, Complejo Hospitalario Universitario de Santiago, Santiago de Compostela, Spain

**Keywords:** Environmental sciences, Health care

## Abstract

Few studies have compared the prevalence of asthma in urban and rural settings or explored the issue of whether these two manifestations of the disease may represent different phenotypes. The aim of this study was: (a) to establish whether the prevalence of asthma differs between rural and urban settings, and b) to identify differences in the clinical presentation of asthma in these two environments. Descriptive epidemiological study involving individuals aged 18 or over from a rural (*n *= 516) and an urban population (*n* = 522). In the first phase, individuals were contacted by letter in order to organize the administration of a first validated questionnaire (Q1) designed to establish the possible prevalence of bronchial asthma. In the second phase, patients who had presented association patterns in the set of variables related to asthma in Q1 completed a second validated questionnaire (Q2), designed to identify the characteristics of asthma. According to Q1, the prevalence of asthma was 15% (*n* = 78) and 11% (*n* = 59) in rural and urban populations respectively. Sixty-five individuals with asthma from the rural population and all 59 individuals from the urban population were contacted and administered the Q2. Thirty-seven per cent of the individuals surveyed had previously been diagnosed with bronchial asthma (35% in the rural population and 40% in the urban setting). In the urban asthmatic population there was a predominance of women, a greater personal history of allergic rhinitis and a family history of allergic rhinitis and/or eczema. Asthma was diagnosed in adulthood in 74.8% of the patients, with no significant differences between the two populations. Regarding symptoms, cough (morning, daytime and night) and expectoration were more frequent in the urban population. The prevalence of asthma does not differ between urban and rural settings. The differences in exposure that characterize each environment may lead to different manifestations of the disease and may also affect its severity.

## Introduction

Bronchial asthma is one of the most prevalent chronic diseases, with more than 350 million affected people in the world^1^. Its prevalence varies between countries^2^ and also between rural and urban areas, although in the latter case the results are inconsistent^3^. In rural areas, it has been proposed that exposure to a greater number of infectious agents and endotoxins from nearby farms may prevent the onset of asthma^4^, especially in children; however, in the adult population these same exposures may aggravate existing asthma^5^. In contrast, in large cities, exposure to smoking and air pollution may predispose to a higher prevalence of asthma^6^. It has also been postulated that the difference between prevalence may be due to differences in accessibility to health resources^3^.

Few studies have compared the prevalence of asthma in urban or rural settings^7–10^, and even fewer have sought to establish whether the clinical presentation differs in these environments and whether they may actually represent two different phenotypes of the disease. Currently, asthma patients tend to be grouped according to whether they present a T2 or a non-T2 response^11^. In general, in the T2 response two phenotypes can be distinguished: one allergic, in which Th2 response mechanisms predominate, and the other eosinophilic, in which the response is mediated by ILC2s^12^. The non-T2 response encompasses patients with neutrophilic inflammation or without apparent inflammation (known as paucigranulocytic asthma)^13^. There are different mechanisms that could explain neutrophilic inflammation in patients with asthma. Some studies have shown a possible activation of the Th17 pathway^14,15^ while others propose a dysregulation of the innate immune response associated with IL-1b or CXCR2^16^. It has also been proposed that in patients in whom bronchial remodeling has led to the appearance of bronchiectasis, bacterial colonization may increase the number of neutrophils in the airways^17^ or that the corticosteroid treatment itself, which reduces the number of eosinophils, facilitates this neutrophilic inflammation^18^. Finally, regardless of whether the response is T2 or non-T2, it has also been postulated that there may be a mixed Th2/TH17 response^19^.

Whether an individual has one type or another of asthma basically depends on the interaction between genetics and the environment to which they are exposed^20^. In this regard, the different exposures to which individuals living in rural or urban areas may be subject may lead to different forms of presentation of asthma. The objective of the present study is twofold: first, to establish whether there are differences in the prevalence of asthma between rural and urban settings and, second, to record any differences in the clinical presentation of the disease in these two environments.

## Methods

### Study population

**Figure 1:**
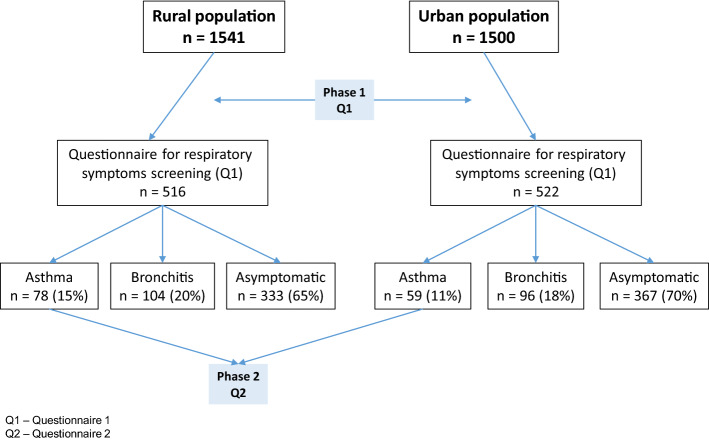
Flow chart of patient enrolment.

One rural and one urban population were studied. The rural population consisted of all individuals over 18 years old living in Ribes de Freser, a mountain town in the Eastern Pyrenees; the group comprised1,760 inhabitants (883 men/877 women) of whom 1,541 were over 18 years of age at the time of the study. The urban population consisted of 1500 randomly selected individuals over 18 years of age from Horta-Guinardó a district in the city of Barcelona with 170,249 inhabitants (Fig. 1). The district of Horta-Guinardó has 11 neighborhoods; for the randomization of the population of this area, 140 questionnaires were introduced at random into the residential mailboxes of each of these neighborhoods to ensure an adequate representation of the area as a whole.

The study was approved by the local Ethics Committee (Hospital Vall d’Hebron Ethics Committee approval PR(AG)367/2011) and all subjects signed informed consent prior to participation. All methods were performed in accordance with the relevant guidelines and regulations.

## Design of the study

### Descriptive, epidemiological study carried out in two phases

#### First phase

A questionnaire for respiratory symptom screening (Q1), previously published by the group^21^, was sent by post to the members of both populations. They were asked to complete it and return it to the investigators by prepaid postage. This questionnaire included questions on symptoms extracted from the ECRHS survey^22^. In the rural population, the town council was responsible for sending the questionnaire to all inhabitants over 18. In the urban setting, 1500 questionnaires were randomly introduced into the mailboxes of homes in the district. Briefly, this earlier study^21^ used multiple correspondence analysis^23^ to assess the association patterns in the set of variables related to respiratory symptoms [(a), (f), (i), (j), (k), (l), and (m)]. Asthma was defined based on an affirmative answer to at least one of the three questions (a) Has a doctor ever told you that you have asthma?, (f) Have you had an asthma attack in the last 12 months? or (m) Have you taken any asthma medication in the last 12 months?. Chronic bronchitis was defined based on a positive response to questions (k) Do you usually cough most days for at least three months of the year? and/or (l) Do you cough up phlegm during at least three months a year? and negative responses to the three asthma questions (a), (f) or (m). Rhinitis was established in the case of a positive answer to questions (c) Has a doctor ever told you that you have rhinitis? and/or (g) Have you had allergic rhinitis in the last 12 months? and dermatitis with a positive answer to questions (b) Has a doctor ever told you that you have dermatitis? and/or (h) Have you had eczema or skin allergies in the last 12 months?.

In the Q1 questionnaire, patients were asked for their consent to participate in the second phase. They were not informed of the main hypothesis of the study, that is, the possible association between environmental exposure and respiratory symptoms.

#### Second phase

The individuals who agreed to participate in this second phase and who were diagnosed with possible bronchial asthma on the basis of the Q1 questionnaire were contacted by telephone and administered a second questionnaire (Q2) designed to identify the characteristics of their asthma. This questionnaire, adapted from the European Community Respiratory Health Survey II (ECRHS II)^24^, focused especially on patients’ general characteristics and symptoms, exposure at work, in the home or in the environment, and the relationship of symptoms with these forms of exposure. The interviews were conducted by pulmonologists who are experts in asthma at the Vall d'Hebron University Hospital.

### Statistical analysis

Categorical variables were expressed as percentages and continuous variables as means (standard deviation). The chi-square calculation was performed for the analysis of the qualitative variables, Student's t-test for the grouped quantitative variables with normal distribution and the Mann–Whitney test for the grouped quantitative variables without normal distribution (the Shapiro–Wilk test was used to determine the normal distribution in the quantitative variables). A two-sided *p* value < 0.05 was considered statistically significant. The statistical program STATA 16 was used for the analyses.

### Results

#### First phase

Five hundred and sixteen individuals from the rural population (Response rate = 33%) and 522 individuals from the urban population (Response rate = 35%) responded to the survey (Fig. 1). Table 1 details the characteristics of both populations. The prevalence of possible asthma in the rural population was 15% (i.e., 78 individuals responded positively to questions “a”, “f” or “m”) and 11% in the urban population (i.e., 59 individuals responded positively to questions “a", "f" or "m") (*p* = 0.320). One hundred and four patients in the rural population were classified as having possible chronic bronchitis (a prevalence of 20%), and 96 in the urban population (a prevalence of 18%) (*p* = 0.215).

**Table 1:** Demographic and clinical characteristics of the study populations (Phase I).

	Total *n* = 1038	Rural *n* = 516	Urban *n* = 522	*p* value
Age, mean (SD)	61 (16.2)	60 (16.8)	61 (15.9)	0.625
Gender (female), *n*	611 (59)	281 (54)	330 (63)	0.420
**Smoking habit**, **n** **(%)**				
Smoker	145 (14)	64 (12)	81 (16)	0.524
Ex-smoker	312 (30)	161 (31)	151 (29)	
Non smoker	581 (56)	291 (57)	290 (55)	
Asthma, yes, *n* (%)*	137 (13)	78 (15)	59 (11)	0.320
Bronchitis, yes, *n* (%)*	200 (19)	104 (20)	96 (18)	0.211
Rhinitis, yes, *n* (%)*	136 (13)	81 (16)	55 (10)	0.090
Dermatitis, yes, *n* (%)*	170 (16)	90 (17)	80 (15)	0.410
**Characteristics of both study regions**				
Climate				
Summer				
Temperature, ºC				
Humidity, %		16–25	20–30	
Winter		75	72	
Temperature, ºC		0–15	5–15	
Humidity, %		67	68	
**Socioeconomically status**				
University studies, %		29	26	
No studies or primary studies, %		21	21	
Index disposable income/inhabitant		100	78	
Access to medical care				
Primary care service		1	11	
Hospital		1**	3**	
Occupational activities		Tourism	Services	
		Livestock		

*Based on positive answers to questions in Q1 (17) **Not exclusive to the area.

No significant differences were found in the variables analyzed between rural and urban individuals in the population classified as asthmatic in Q1 (Table 2).

**Table 2:** General characteristics of the population with asthma symptoms according to the Q1 questionnaire.

	Rural *n* = 78	Urban *n* = 59	*p* value
**Demographic characteristics**			
Gender (female), *n* (%)	43 (55)	41 (69)	0.384
Age, mean (SD)	59 (19)	58 (21)	0.519
**Smoking habit**			
Non-smoker, *n* (%)	44 (56)	54 (53)	0.210
Smoker	9 (12)	17 (18)	
Ex-smoker	25 (32)	29 (25)	
**Allergic history**			
Rhinitis, *n* (%)	29 (37)	17 (29)	0.239
Dermatitis, *n* (%)	24 (31)	21 (35)	0.839

*SD* Standard deviation.

#### Second phase

The second survey was administered to 65 of the 78 individuals (83.3%) classified as asthmatic in the rural population in the first survey and to 50 of the 59 individuals (84.7%) of the urban population. Seven individuals (three rural) did not provide correct data and it was not possible to contact them. Twelve (five rural) refused to continue in the study and three (two rural) had died by the time of contact. Table 3 shows the general characteristics of the population finally included. In all, 37% of the individuals surveyed had previously been diagnosed with bronchial asthma (35% in the rural population and 40% in the urban). In the urban asthmatic population there was a predominance of women, more personal history of allergic rhinitis and more family history of allergic rhinitis and/or eczema; urban dwellers with asthma also presented a greater personal history of severe respiratory infection during childhood, were more likely to live either currently or during childhood with family members who smoke, and comprised a greater number of active smokers. Patients in this population also presented more symptoms in winter, used asthma control medication more frequently, had required a greater number of emergency room visits due to respiratory problems, and presented a greater number of exacerbations in the last year. Asthma had been diagnosed in adulthood in 74.8% of the patients, with the mean age of onset of symptoms being 44 years; there were no significant differences in this regard between the two populations.

**Table 3:** General characteristics of asthmatic individuals included according to Q1.

	Total *n* = 115	Rural *n* = 65	Urban *n* = 50	*p* value
**Demographic characteristics**				
Gender (female), *n* (%)	71 (62)	33 (51)	38 (76)	**0.006**
Age, mean (SD)	60.7 (15.5)	60.5 (15.3)	60.9 (15.9)	0.826
Caucasian, *n* (%)	102 (89)	62 (95)	40 (80)	**0.010**
**Smoking habit**				
Non-smoker, *n* (%)	54 (47)	27 (41)	27 (54)	**0.042**
Smoker	19 (16)	8 (12)	11 (22)	
Ex-smoker	42 (36)	30 (46)	12 (24)	
Age of onset, mean (SD)	17.1 (3.4)	17.1 (4.0)	17.2 (2.3)	0.881
Smoking cessation age, mean (SD)	41.9 (14.3)	40.6 (15.2)	45.2 (11.6)	0.355
Paq/year, mean (SD)	22.2 (18.9)	24.1 (22.9)	19.3 (10.1)	0.346
Passive smoker, yes, *n* (%)	37 (32)	8 (12)	29 (68)	**0.001**
Passive smoker in childhood, yes, *n* (%)	84 (73)	42 (65)	42 (84)	**0.020**
**Asthma diagnosis**				
Previous asthma diagnosis, *n* (%)	43 (34)	23 (35)	20 (40)	0.612
Age of first asthma attack, mean (SD)	27.5 (18.5)	28.9 (20.7)	25.8 (16.1)	0.770
**Season with most symptoms,** **n** **(%)**				
None	3 (7)	3 (13)	0	**0.018**
Spring	24 (56)	16 (70)	8 (40)	
Summer	2 (5)	0	2 (10)	
Autumn	0	0	0	
Winter	14 (33)	4 (17)	10 (50)	
**Allergy/asthma history**				
Asthmatic first-degree relatives, *n* (%)	52 (45)	27 (41)	25 (50)	0.366
First-degree relatives with allergic rhinitis and/or eczema, *n* (%)	31 (27)	11 (17)	20 (40)	**0.006**
Severe respiratory infection in childhood, *n* (%)	32 (28)	12 (18)	20 (40)	**0.011**
Allergic rhinitis, *n* (%)	58 (50)	26 (40)	32 (64)	**0.001**
Atopic dermatitis, *n* (%)	42 (36)	23 (35)	19 (38)	0.773
Allergy to insect venom, *n* (%)	18 (16)	13 (20)	5 (10)	0.143
	16 (90)	11 (85)	5 (10)	0.352
Local				
Systemic (dyspnea)	2 (11)	2 (15)	0	
Drug allergy (dyspnea), *n* (%)	9 (8)	4 (6)	5 (10)	0.446
Adult-onset asthma > 18 yrs, n (%)	74 (75)	43 (77)	31 (72)	0.594
Age of asthma onset, mean (SD)	44.6 (15.3)	43.6 (16.7)	46.0 (13.2)	0.429
**Medication and exacerbations, last year**				
Current medication use, *n* (%)	28 (65)	11 (48)	17 (85)	**0.011**
Use of inhalers, *n* (%)	57 (49)	29 (45)	28 (56)	0.226
Oral medication for dyspnea, *n* (%)	22 (19)	9 (14)	13 (26)	0.100
Injectable medication for dyspnea, *n* (%)	6 (5)	3 (5)	3 (6)	0.674
Asthma exacerbations, mean (SD)	0.4 (0.5)	0.3 (0.5)	0.6 (0.5)	**0.040**
Nº Emergency room visits last year, mean (SD)	0.4 (1.2)	0.1 (0.2)	1.01 (1.7)	**0.001**
Nº hospitalizations last year, mean (SD)	0.2 (0.5)	0.1 (0.4)	0.3 (0.6)	0.524

*SD* Standard deviation.

Significant values are in [bold].

The most prevalent symptoms related to asthma were wheezing (58.3%), exertional dyspnea (54.8%), morning cough (40%), night cough (39.1%), and morning expectoration (31.3%) in both populations. No significant differences were observed in symptoms between the populations except in cough (morning, daytime and night) and expectoration, which were more frequent in the urban population. The percentage of patients with continuous symptoms was also higher in the urban population (Table 4).

**Table 4:** Respiratory symptoms of asthmatic individuals included according to Q1.

	Total *n* = 115	Rural *n* = 65	Urban *n* = 50	*p* value
**Wheezing, *n*** **(%)**	67 (58)	38 (58)	29 (58)	0.960
Wheezing with associated dyspnea	44 (68)	26 (68)	18 (62)	0.857
Wheezing without respiratory infection	43 (64)	24 (63)	19 (65)	0.842
Chest tightness, *n* (%)	18 (16)	8 (12)	10 (20)	
Resting dyspnea, *n* (%)	24 (21)	16 (25)	8 (16)	
Exertional dyspnea	63 (55)	36 (55)	27 (54)	
Paroxysmal nocturnal dyspnea	21 (18)	11 (17)	10 (20)	
Dyspnea according to mMRC***, **n** **(%)**				
mMRC 0	42 (41)	19 (33)	22 (50)	0.260
mMRC 1	39 (39)	25 (44)	14 (32)	0.260
mMRC 2	16 (16)	10 (17)	6 (14)	0.882
mMRC 3	5 (5)	3 (5)	2 (5)	0.672
mMRC 4	0	0	0	0.408
Age of onset of symptoms, mean (SD)	41.2 (20.3)	38.4 (20.3)	44.9 (19.9)	0.147
Night cough attacks, *n* (%)	45 (39)	23 (35)	22 (44)	0.348
Morning cough, *n* (%)	46 (40)	12 (18)	34 (68)	**0.001**
**Day or night cough,** **n** **(%)**	29 (25)	6 (9)	23 (46)	**0.001**
Chronic cough*	10 (19)	0	10 (29)	**0.014**
Age of onset of chronic cough, mean (SD)	41.3 (15.6)	44.6 (12.4)	39.9 (16.7)	0.329
**Morning expectoration, yes, n (%)**	36 (31)	14 (21)	22 (44)	**0.010**
Chronic bronchitis**, yes, *n* (%)	19 (53)	9 (64)	10 (45)	0.270
Age of onset of chronic bronchitis, mean (SD)	41.9 (17.1)	43.5 (18.1)	40.9 (16.5)	0.660
**Frequency of respiratory symptoms, n** **(%)**				
Never	41 (36)	29 (45)	12 (24)	**0.001**
Only on rare occasions	32 (28)	30 (46)	2 (4)	
Repeatedly	27 (23)	6 (9)	21 (42)	
Continuously	15 (13)	0	15 (30)	
Non-cardiopulmonary walking inability, *n* (%)	14 (12)	8 (12)	6 (12)	0.960

*Chronic cough duration longer than three months; **Chronic bronchitis Cough and expectoration lasting more than three months for two years in a row; ***mMRC Modified Medical Research Council. *SD *Standard deviation.

Significant values are in [bold].

Regarding occupational, domestic and environmental exposure (Table 5), 45.2% of individuals were working at the time of the interview. Occupational exposures that might affect respiratory health were recorded in 55% of the rural population and in 40% of the urban population (*p* = 0078). Twenty-nine per cent were exposed to smoke and dust; 17.4% related their asthma symptoms to work and 7.8% had had to change their job for this reason. These events were more frequent in the rural population. Symptoms due to contact with animals and/or dust were reported by 45% of the study population, and were more frequent in the urban setting. Symptoms due to contact with pollen and/or in parks were recorded by 53% of respondents; 39.1% described symptoms when being near irritating odors (bleaches, perfumes, gasoline, etc.) and 33.9% reported symptoms when noticing a subjective increase in environmental pollution. The exposure to irritants and environmental pollution generated more coughing, nasal congestion and eye irritation in the urban population, and more dyspnea in the rural population.

**Table 5:** Occupational and domestic exposure in the study population.

	Total	Rural	Urban	*p* value
	*n* = 115	*n* = 65	*n* = 50	
**Occupational context**				
Currently working, yes, *n* (%)	52 (45)	27 (41)	25 (50)	0.366
Respiratory symptoms at work, yes, *n* (%)	20 (17)	12 (18)	8 (16)	0.73
Change jobs due to these symptoms	9 (8)	9 (14)	0	**0.006**
Current job, *n* (%)				
Occupational exposures that might affect respiratory health, yes		36 (55)	20 (40)	0.078
Exposure to dust/fumes, yes, *n* (%)	33 (29)	26 (40)	7 (14)	**0.003**
**Exposure to animals**				
Current contact with animals, yes, *n* (%)	35 (34)	22 (34)	13 (26)	0.365
Time of coexistence with animals, years, mean (SD)	9.1 (10.0)	10.8 (11.9)	6.4 (4.6)	0.217
Contact with animals in childhood, yes, n (%)	69 (63)	36 (56)	33 (71.4)	0.097
Time of coexistence with animals, years, mean (SD)	10.1 (4.5)	10.9 (4.8)	9.8 (4.5)	0.478
**Place of residence**				
Years living at current address, mean (SD)	31.9 (21.0)	30.0 (21.2)	34.2 (20.8)	0.276
Previous other residences, yes, *n* (%)	76 (66)	41 (63)	35 (70)	0.437
Number of years living at the previous address, mean (SD)	24.1 (15.2)	23.5 (17.1)	24.8 (12.8)	0.723
**Symptoms and environmental exposure**				
**Symptoms of contact with animals and/or dust, yes, n (%)**	52 (45)	30 (46)	22 (44)	0.818
Cough	17 (33)	6 (20)	11 (50)	**0.023**
Panting	1 (2)	1 (3)	0	0.387
Chest tightness	4 (8)	2 (7)	2 (9)	0.746
Dyspnea	14 (27)	10 (33)	4 (18)	0.224
Nasal congestion	48 (92)	27 (90)	21 (95)	0.466
Eye congestion	35 (67)	16 (53)	19 (86)	**0.012**
**Symptoms of contact with pollen and/or in parks, yes, n (%)**	61 (53)	35 (54)	26 (52)	0.884
Cough	23 (38)	14 (40)	9 (35)	0.668
Panting	2 (3)	0	2 (8)	0.095
Chest tightness	4 (7)	1 (3)	3 (11)	0.176
Dyspnea	15 (25)	11 (31)	4 (15)	0.15
Nasal congestion	51 (84)	27 (77)	24 (92)	0.114
Eye congestion	45 (74)	24 (69)	21 (81)	0.284
**Symptoms near inhaled irritants, yes, n (%)**	45 (39)	25 (38)	20 (40)	0.867
Cough	22 (49)	7 (28)	15 (75)	**0.002**
Panting	1 (2)	0	1 (5)	0.258
Chest tightness	4 (9)	3 (12)	1 (5)	0.412
Dyspnea	23 (51)	20 (80)	3 (15)	**0.001**
Nasal congestion	26 (58)	10 (40)	16 (80)	**0.007**
Eye congestion	19 (42)	6 (24)	13 (65)	**0.006**
**Symptoms of exposure to environmental pollution, yes, n (%)**	39 (34)	20 (31)	19 (38)	0.417
Cough	15 (38)	4 (20)	11 (58)	**0.015**
Panting	2 (5)	0	2 (10)	0.136
Chest tightness	3 (8)	3 (15)	0	0.079
Dyspnea	17 (44)	15 (75)	2 (10)	**0.001**
Nasal congestion	19 (49)	4 (20)	15 (79)	**0.001**
Eye congestion	15 (38)	3 (15)	12 (63)	**0.002**

*SD* Standard deviation.

Significant values are in [bold].

## Discussion

The results of this study do not show differences in the prevalence of asthma between urban and rural areas, but they do show differences in the characteristics of asthma and probably also in its severity. The most relevant findings were the following: there was a predominance of women with asthma in the urban setting; urban asthma sufferers presented more allergic symptoms in contact with allergens than their rural counterparts; their major symptoms were cough, rhinitis and eye irritation; they required more treatment, presented more exacerbations and made more emergency room visits for respiratory problems than asthmatics in the rural population.

The objective of the current study is to establish whether there are differences in the prevalence of asthma between urban and rural areas and, if so, to identify the factors that cause them. It has been demonstrated that exposure to a microbial environment in early childhood, typical of rural environments, may play a role in the subsequent development of asthma. Based on data from a subpopulation of The European Community Respiratory Health Survey (ECRHS), Timm et al. ^25^ reported a prevalence of asthma of 8% in individuals who lived near farms and one of 11% in those who lived in city centers in a northern European population. They also established an urban–rural gradient of asthma, according to which subjects growing up on a livestock farm had significantly less late-onset asthma than subjects growing up in cities. In contrast, a greater exposure to environmental pollutants might explain the higher incidence of asthma in individuals who live in cities, especially in city centers^26^. However, even though one recent systematic review of 70 articles established that the prevalence of asthma seemed to be higher in urban than in rural areas^27^, it is difficult to reach firm conclusions: most of the studies carried out are very heterogeneous terms of design, the definition of the condition, and the environmental exposures described, and very few studies take into account the possible underdiagnoses of asthma in rural areas due to logistical reasons^3^. Indeed, in our study, only 35% of possible asthmatics in the rural population had previously been diagnosed with the disease; what is more, the studies that do not show differences or report a greater risk of asthma to the rural population are the ones carried out more recently^27–29^. Finally, a recent study establishes that exposure even to low doses of pollutants indoors could equalize the incidence of asthma in children between rural and urban areas^30^.

Another possible reason for the differences observed in the prevalence of asthma between rural and urban populations, and which by itself could be the object of study hypotheses, is whether urban and rural asthma represent different phenotypes of the disease. This issue has received little attention, but there are grounds to think that it may indeed be the case. As noted above, whether an individual presents one type of asthma or another basically depends on the interaction between genetics and the environment in which he/she is exposed^20^. In this regard, there is evidence in the field of occupational asthma that exposure to high or low molecular weight agents generates different clinical phenotypes of the disease without there being relevant inflammatory changes between the two types of exposure^31^.

Among the differences observed in the present study, we found that urban patients had more allergic rhinitis, more family history of allergic rhinitis and/or eczema, and more asthma symptoms with exposure to aeroallergens. These findings may be conditioned by the different exposures to which individuals are subjected in rural and urban settings^9^. In fact, although the theory of hygiene cannot explain differences in the prevalence of asthma, it can account for the different levels of awareness between the rural and urban populations^32^. Furthermore, the association of aeroallergens with city-specific environmental pollutants can contribute to exacerbating asthma, as our group has recently shown^19^. Although these observations do not necessarily reflect differences in the prevalence of asthma, they show that the asthma suffered by individuals in rural or urban areas is different.

It is also interesting that urban asthmatic patients presented more cough, both as a base symptom and when exposed to allergens, irritants, or environmental pollutants than the rural population. To be able to explain this finding, further studies are probably necessary in order to determine whether there are differences in terms of lung function or distinctive types of bronchial inflammation between the two populations. The characteristics of this study do not allow us to establish the actual cause, although it is known that greater bronchial obstruction is more often associated with the presence of cough and a greater degree of bronchial hyperresponsiveness to wheezing and thickness^33^. Nor, based on the results obtained, can we establish with certainty whether in fact cough is a feature that differentiates between the two types of asthma or is merely a finding that could be explained by a confounding factor such as tobacco exposure. Indeed, in the urban asthma population there may be greater exposure (both active and passive) to tobacco smoke, while in the rural population ex-smokers predominate. However, the fact that, in the first phase of the study, the diagnosis of chronic bronchitis was more frequent in the rural population, and that no differences were found when informants were specifically asked about chronic bronchitis in the second phase, would argue against this possibility. The relationship between sex and the consequences of smoking also raises doubts since it has been shown that female smokers and ex-smokers in rural areas are more likely to be diagnosed with asthma than non-smoking urban women^34^, especially taking into account that the proportion of women with asthma was higher in our urban population than in our rural population. Likewise, the possible relationship between asthma and/or asthma symptoms in patients with exposure to secondhand smoke has also received little attention. A cross-sectional study using the Canadian National Population Health data, collected from 1994 to 2000, showed a higher prevalence of asthma among smokers and nonsmokers in urban than in rural residents. Higher stress levels and the lack of open spaces compared with their rural counterparts, may be reasons for this higher prevalence of asthma among smokers living in urban areas, while among nonsmokers in urban areas the reasons may be environmental factors and exposure to secondhand smoke^35^.

Asthma exacerbations have also been shown to be a differential factor between urban and rural asthma. The fact, for example, that rural asthmatic patients may present a higher incidence of exacerbations in spring could be related to a greater exposure to allergens in this season, while the greater number of exacerbations in winter in urban asthmatics might be due to a greater exposure to indoor pollutants caused by a decrease in air circulation between outdoor and indoor environments as windows tend to be closed at this time of year^26,30^. However, more relevant is the fact that patients with urban asthma had made more visits to the emergency room for respiratory problems and presented more exacerbations in the last year. In this connection, Smith et al.^36^ conducted a cross-sectional study in the US exploring the risk factors associated with healthcare utilization among 3,013 Arizona Medicaid patients with asthma. These authors observed that urban areas had higher rates of asthma-related hospital visits compared to rural counties, and that rates were higher in adults than in adolescents. Furthermore, several authors have pointed out that urban asthma may be associated with greater morbidity than rural asthma ^20^^,^^25^^,^^26^^,^^28^, and although these results may be affected by differences in accessibility to the health system in the two areas^3^, it is generally agreed that exposure to environmental pollutants, more typical of urban areas, may well increase the number of exacerbations in these patients^37^.

One of the most important limitations of the study is the low response rate (around 35%) in the first phase. However, the absolute number of responses obtained, close to 600 individuals in each population, probably validates the results obtained. Another limitation, inherent in all epidemiological studies, is the definition of asthma itself. In this regard, we decided to use the results obtained from a correspondence analysis from the first questionnaire previously published by the group, and which has demonstrated its validity^21^. Finally, the study design did not allow us to establish possible risk factors that might increase the differences observed between urban and rural asthma.

In conclusion, the results of this study establish two possible working hypotheses for future work: first, that the prevalence of asthma does not necessarily differ between urban and rural settings and, second, that the different characteristic exposures of each environment may lead to different manifestations of asthma and to different degrees of disease severity, as has already been shown, for example, in occupational asthma. Clinical, lung function and bronchial inflammation studies are needed to confirm that urban and rural asthma may actually be two different asthma phenotypes.

## Data Availability

All data generated or analysed during this study are included in this published article.
